# *HOXC8* mediates osteopontin expression in gastric cancer cells

**DOI:** 10.7150/jca.84460

**Published:** 2023-08-21

**Authors:** Chung-Yu Tsai, Jia‑Bin Liao, Yi-Chen Lee, Yi-Fang Yang

**Affiliations:** 1 Division of General Surgery, Department of Surgery, Kaohsiung Veterans General Hospital, Kaohsiung, Taiwan.; 2 Department of Pathology and Laboratory Medicine, Kaohsiung Veterans General Hospital, Kaohsiung, Taiwan.; 3 Department of Anatomy, School of Medicine, College of Medicine, Kaohsiung Medical University, Kaohsiung, Taiwan.; 4 Department of Medical Education and Research, Kaohsiung Veterans General Hospital, Kaohsiung, Taiwan.

**Keywords:** *HOXC8*, osteopontin, *SPP1*, and gastric cancer

## Abstract

Genes of the homeobox (HOX) family encode transcription factors, which play a role in cancer progression. However, their role in gastric cancer has not been adequately evaluated. Herein, we evaluated the genetic changes and mRNA of target genes of the HOX family in gastric cancer patients using publicly available online datasets. We found that *HOXC8* was amplified in gastric cancer tissues, and mRNA expression levels were significantly associated with tumor status (*P*=0.044) and poor overall survival (*P*<0.01). *HOXC8* knockdown significantly reduced the viability of gastric cancer cell lines. *HOXC8* modulated the expression of secreted phosphoprotein 1 (SPP1, osteopontin) and phosphorylation of AKT/ERK in gastric cancer cells. Survival analysis demonstrated a decrease in overall survival rates among the high *HOXC8*/high* SPP1* expression group compared with the low *HOXC8*/low* SPP1* expression group. In conclusion, *HOXC8* may be an independent prognostic factor and serve as a useful predictive biomarker for gastric cancer.

## Introduction

The survival and prognosis of cancer patients are associated with stage status development. Gastric cancer is the sixth most common malignant cancer (1.09 million cases in 2020, World Health Organization) and the leading cause of cancer-related death in the world 1-4. Among the deaths in Taiwan in 2020, 50,161 were due to cancers, 2,339 (5.2%) of which occurred from gastric cancer (from the Ministry of Health and Welfare of Taiwan). Unfortunately, the lack of highly sensitive and specific biomarkers to diagnose gastric cancer leads to most patients being in the advanced stage before diagnosis 5-7. Therefore, investigating and improving the diagnostic sensitivity of cancer biomarkers for early-stage tumors is beneficial for improving the survival rate of gastric patients.

The human homeobox (HOX) genes have four chromosomal clusters and 39 HOX genes: *HOXA* (chromosome 7), *HOXB* (chromosome 17), *HOXC* (chromosome 12), and *HOXD* (chromosome 2) 8. The HOX genes encode transcription factors that are involved in a number of biological processes, including embryonic development, cell proliferation, and differentiation, among others 9, 10. There has been substantial evidence of aberrant *HOX* gene expression in multiple cancers such as prostate, pancreatic, lung, and breast cancer 8, 11-13. Numerous studies have shown that diverse HOX genes can either enhance or inhibit the cancer progression process on their abnormal expression in different organs. For example, the HOXB gene is a prognostic factor in breast cancer 14, HOXA13 expression impaired the chemotherapy in gastric cancer cells 15, HOXB5 promotes metastasis in hepatocellular carcinoma 16 and HOXA13, HOXD13, and HOXC6 were promoted colorectal cancer 17-19. Furthermore, lymphoblastic leukemia with mixed-lineage leukemia translocations shows increased expression of *HOXA4*, *HOXA5*, *HOXA7*, *HOXA9,* and *HOXB5*, which is related to poor prognosis 20-24. *HOXC4-6* and *HOXC8* are upregulated in primary tumor and cancer cell lines and are not normally expressed in normal prostate tissues or cell lines 25, 26. HOX protein can modulate other proteins to enhance or repress gene expression 27 and it also can be expressed in cancer stem cells 28. In gastric cancer and HOXC6 promotes invasion ability 29. However, downstream targets of HOX genes have still not been fully identified.

Secreted phosphoprotein 1 (SPP1), also known as osteopontin (OPN) protein was upregulated in primary and metastatic lesions of gastric cancer, which indicates that OPN may play a role in gastric cancer 30, 31. OPN was first described as a glyco-phosphoprotein that is secreted from malignant epithelial cells 32. The functions of OPN in cancer progression, include cell adhesion, chemotaxis, invasion, migration, and the anchorage-independent growth of tumor cells 33. A previous study showed that OPN was downregulated in *HOXC8-*silenced cells 11. In our study, the highest expression of *HOXC8* was observed in gastric cancer tissues. However, the mechanism by which *HOXC8* modulates OPN expression and regulates the malignant phenotype of gastric cancer cells has not been clearly elucidated.

## Materials and Methods

### *In silico* mRNA profiles and Kaplan-Meier analysis of *HOXC8*

*HOXC8* mRNA expression in stomach adenocarcinoma tissues was determined by using The Cancer Genome Atlas (TCGA) dataset and TNMplot (https://tnmplot.com/analysis/). Kaplan-Meier analysis (overall survival) was performed using a publicly available gastric cancer microarray dataset (http://kmplot.com/analysis/).

### Cell lines and culture conditions

TSGH, AZ521, and HR cell lines (human gastric carcinoma) from Dr. Kuo-Wang Tsai at Taipei Tzu Chi Hospital in Taiwan. Cells were cultured in Dulbecco's Modified Eagle Medium with 10% fetal bovine serum and 1% penicillin-streptomycin-glutamine.

### Lentivirus infection

Lentivirus vector control (pLKO-1-shLuc967) and shHOXC8 shRNA viral supernatant (TRCN0000019564, TRCN0000019565) were purchased from the National RNAi Core Facility (Taipei, Taiwan). The viral supernatants were used to infect AZ521 or HR cell lines with 8 μg/mL of polybrene at 72 h. After infection, cells were selected by using 2 μg/mL of puromycin.

### Real-time-polymerase chain reaction (PCR)

Total RNA was extracted using TRI Reagent (Sigma-Aldrich, #T9424), cDNA was synthesized by TaKaRa PrimeScript™ RT reagent Kit (Cat. #RR037A), and PCR reactions were conducted using SYBR system (PCR Biosystems qPCRBIO SyGreen Mix Lo-ROX). The following primer sequences were used: *HOXC8*, forward: 5'TCAAAACTCGTCTCCCAGCC-3'; *HOXC8*, reverse: 5' TTCCAAGGTCTGATACCGGC-3'; *SPP1*, forward: 5'ATGATGGCCGAGGTGATAGTG; *SPP1*, reverse: GAGGTGATGTCCTCGTCTGTAGC; 5'*ACTB*, forward: 5' AGAAAATCTGGCACCACACC-3' and *ACTB*, reverse: 5' AGAGGCGTACAGGGATAGCA-3'.

### Growth curve assay

Cells (2,000 cells/well for AZ-521/shluc, AZ-521/shHOXC8-1, AZ-521/shHOXC8-2, HR/shluc, HR/shHOXC8-1, and HR/shHOXC8-2) were seeded in a 96-well plate for 24-72 h (incubated at 37°C with 5% CO_2_). The cell growth curve was determined using a 3-(4,5-dimethylthiazol-2-yl)-2,5-diphenyltetrazolium bromide assay.

### Colony formation assay

The stable lines (1,000 cells/well) were plated in a 6-well plate. After a 7-day incubation at 37°C with 5% CO_2_, the cells were fixed and stained using crystal violet. The number of colonies was counted using the NIH Image J software.

### Western blot analysis

Western blot analysis was performed using the following primary antibodies: anti-OPN (IBL, #18625,1:1000), anti-pAKT (Cell Signaling, #4060, 1:1000), anti-AKT (Cell Signaling, #4691,1:1000), anti-pERK (Cell Signaling, #9101,1:1000), anti-ERK (Cell Signaling, #9102,1:1000), and β-actin (Sigma-Aldrich, #A5441, 1:5000).

## Results

### *In silico* genetic alterations of *HOXC* family members in stomach adenocarcinoma

**Figure 1:**
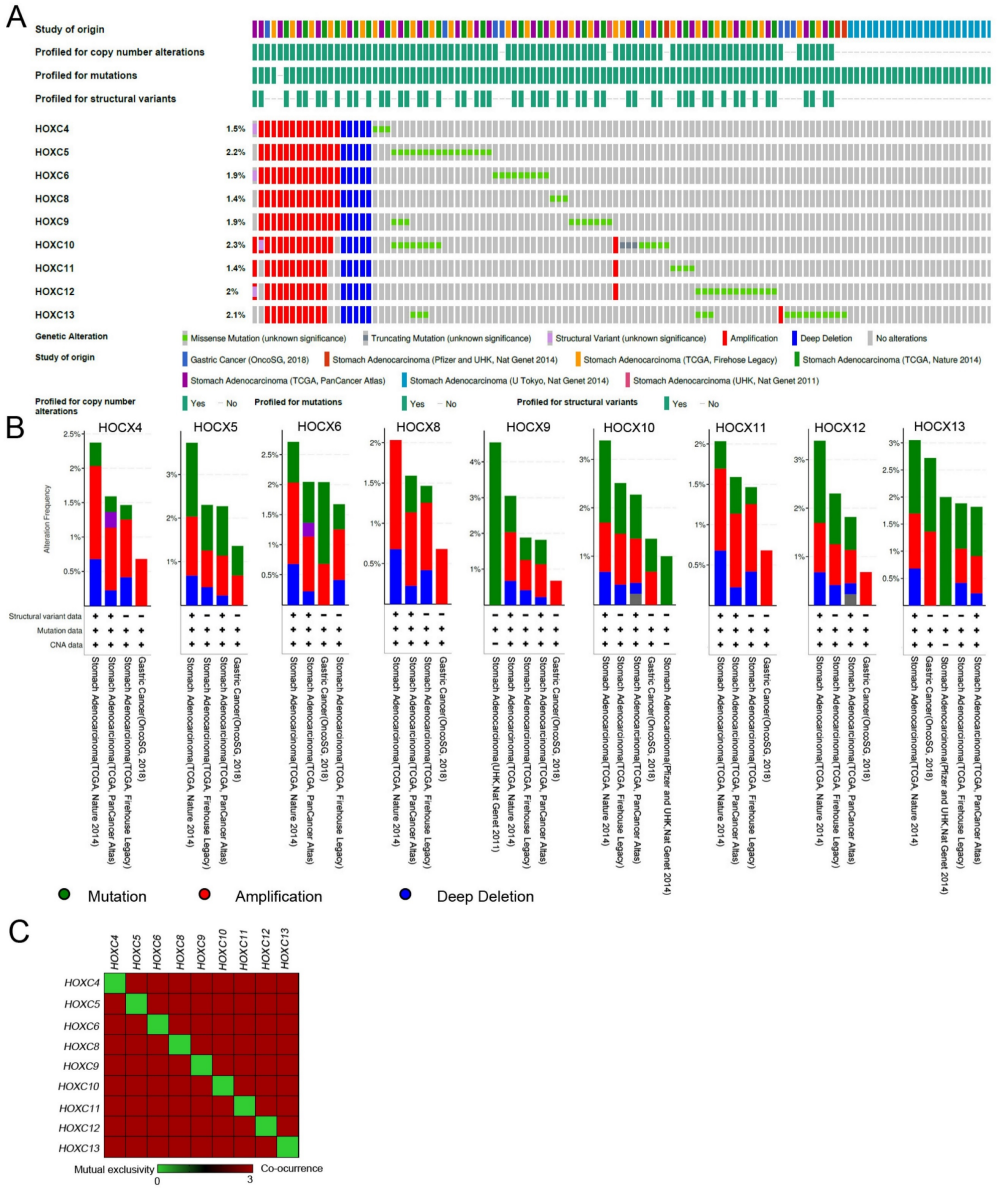
HOXC family amplification in stomach adenocarcinoma (STAD). (A) Oncoprint showing *HOXC4*, *HOXC5*, *HOXC6*, *HOXC8*, *HOXC9*, *HOXC10*, *HOXC11*, *HOXC12*, and *HOXC13* in STAD patients. (B) Cancer type summary of *HOXC4*, *HOXC5*, *HOXC6*, *HOXC8*, *HOXC9*, *HOXC10*, *HOXC11*, *HOXC12*, and *HOXC13* by different STAD cohorts. (C) Co-occurrence analysis of *HOXC* family members in STAD tumors.

cBioPortal was used to examine the genetic alterations of the *HOXC* family in stomach adenocarcinoma (STAD) patients. *HOXC4*~*HOXC13* had a 1.5-2.3% amplification, and *HOXC9* had a 4% mutation ratio in STAD patients (Figure 1A-B). Further analyzing the frequency of co-occurrence of the *HOXC* family genetically altered in the same tumor tissues. Co-occurrence analysis of the STAD sample identified the *HOXC* family genetically altered significant co-occurrence in the same tumor sample (Figure 1C and Table 1). Previous studies showed several members of the *HOXC* family have been well-investigated for their role in gastric cancer 34, 35. Moreover, *HOXC8* knockdown reduced cell proliferation in gastric cancer 36. However, the molecular mechanism of its action remains unclear. Therefore, in this study, we focused on *HOXC8* to further investigate gastric cancer.

### *The HOXC8* expression level was significantly increased and correlates with poor survival rates in STAD patients

**Figure 2:**
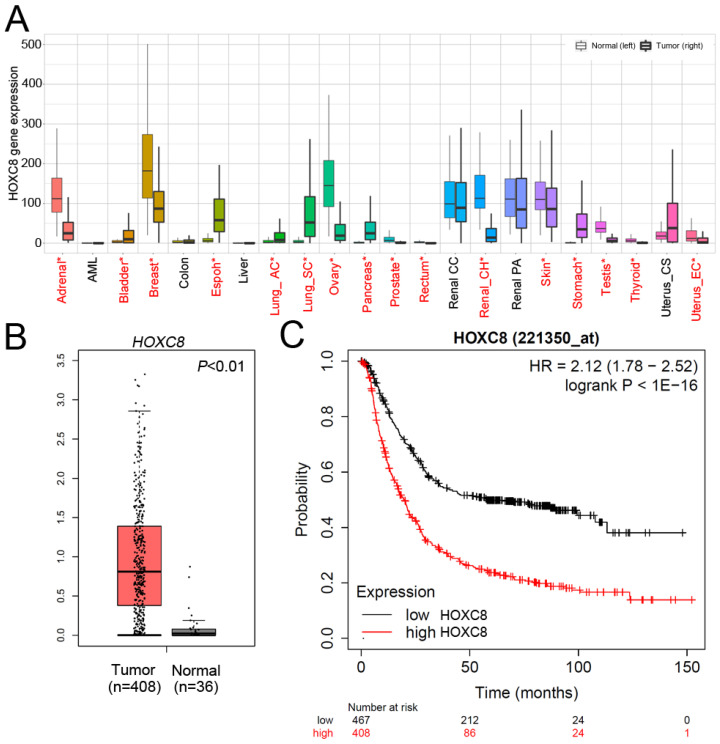
*HOXC8* is overexpressed in gastric cancer and is correlated with poor survival. (A) *HOXC8* mRNA expression in multiple cancer types (TNMplot). (B) The expression of *HOXC8* is significantly upregulated in STAD tumors compared to that in adjacent normal tissue, as determined using TCGA dataset. (C) Kaplan-Meier graph of overall survival in publicly available gastric cancer microarray datasets, stratified according to *HOXC8* expression (Kaplan-Meier Plotter).

We next evaluated the mRNA levels of *HOXC8* in multiple cancer types using TNMplot, which showed that *HOXC8* was upregulated in many cancer types, including STAD (Figure 2A). We further analyzed the TCGA dataset to understand the clinical prognostic value of *HOXC8* in STAD patients. Expression profiles were downloaded from TCGA, including 408 STAD tissues and 36 adjacent normal tissues. As shown in Figure 2B, the expression level of *HOXC8* was significantly increased in STAD patients compared to adjacent normal tissues. The expression levels of *HOXC8* in human STAD tissues were further evaluated in 95 patients with stage I-II STAD and 272 with stage III-IV STAD. *HOXC8* expression analysis was based on the expression of *HOXC8* mRNA. Using the clarified expression criteria, we classified patients into the *HOXC8*-low and *HOXC8*-high groups. The data showed that *HOXC8* expression was significantly associated with tumor stage (*P*=0.044) (Table 2). Next, we detected the overall survival rate of *HOXC8* in a clinical cohort using Kaplan-Meier analysis. A high level of *HOXC8* expression correlated with the overall survival rate in patients with gastric cancer (Figure 2C). However, the detailed role of *HOXC8* in STAD progression remains unclear.

### *HOXC8* knockdown reduced proliferation and colony formation in gastric cancer cells

**Figure 3:**
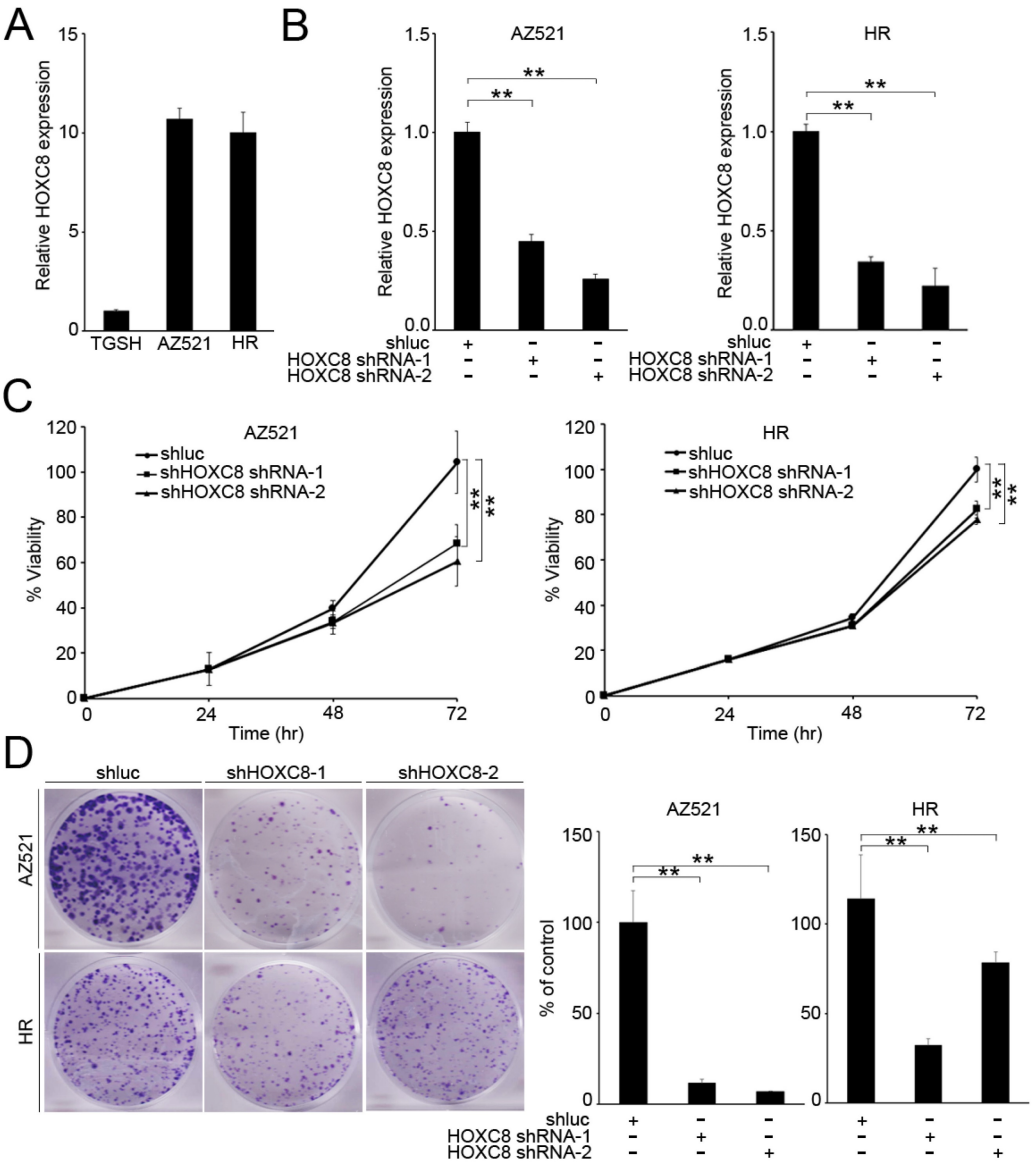
*HOXC8* knockdown inhibits cell proliferation. (A) Real-time polymerase chain reaction (PCR) analysis of *HOXC8* expression in gastric cancer cell lines. (B) Real-time PCR analysis of *HOXC8* expression after infection with shRNA. (C) Effect of *HOXC8* knockdown on proliferation of AZ521 and HR cell lines. (D) Downregulation of *HOXC8* reduced colony formation of gastric cancer cells.

According to the clinical data, we found that *HOXC8* expression was associated with tumor size (Table 2). Therefore, we evaluated the effect of HOXC8 on proliferation and colony formation in gastric cancer cells. First, we examined the expression levels of *HOXC8* in gastric cancer cell lines. The results revealed that *HOXC8* was upregulated in AZ521 and HR cancer cell lines compared with that in a TGCH cell line (Figure 3A). Next, we evaluated the effect of *HOXC8* on cell viability and colony formation in gastric cancer cells. *HOXC8* knockdown significantly reduced the cell growth and colony formation in AZ521 and HR cells (Figure 3B-D).

### *HOXC8* downregulated the inhibitory OPN pathway in gastric cancer cells

**Figure 4:**
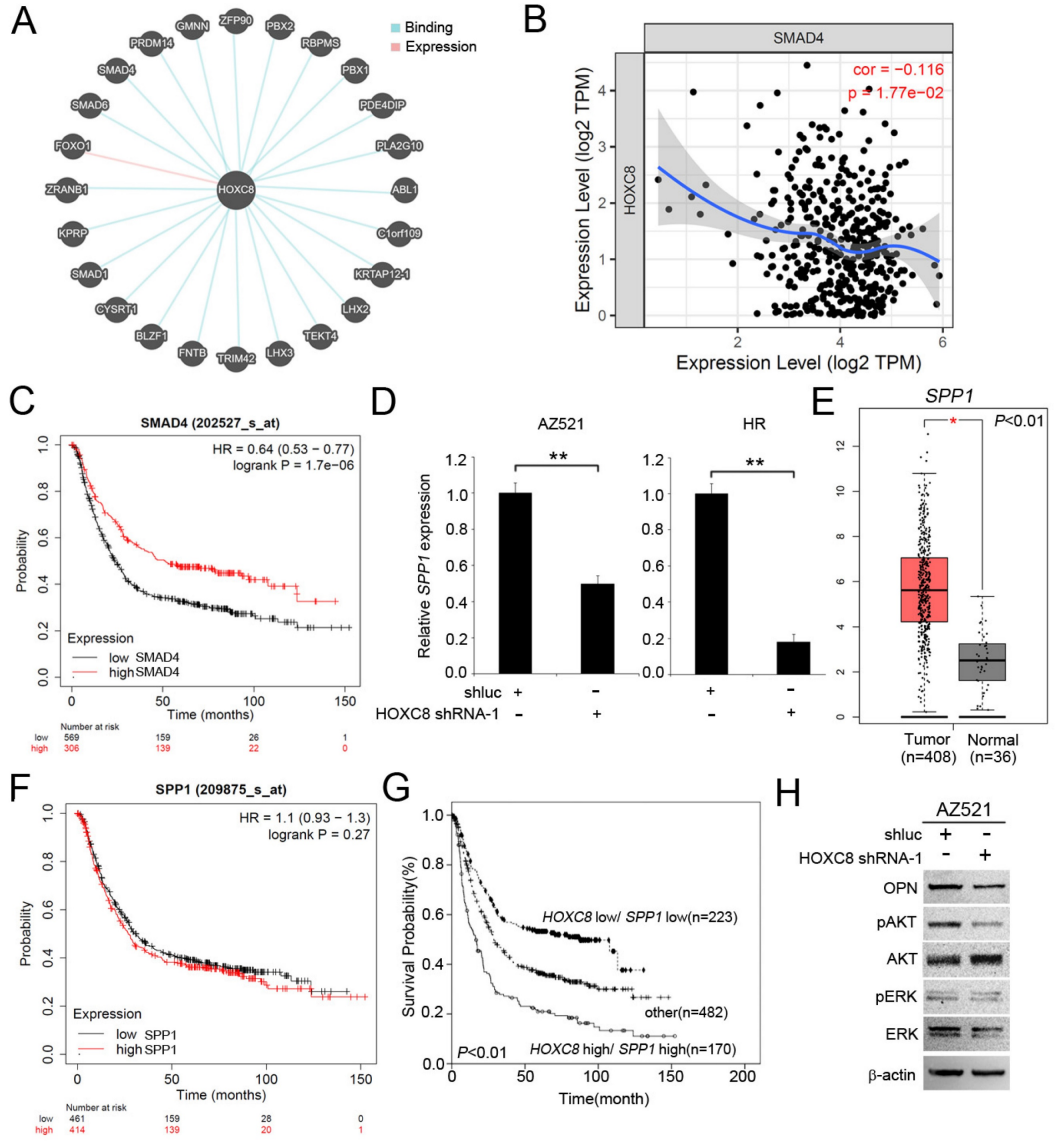
*HOXC8* mediates the OPN pathway in gastric cancer cells. (A) Putative binding interaction targets of HOXC8 were identified from Pathway Commons. (B) Analysis of the correlation between *HOXC8* and *SMAD4* mRNA expression by using TIMER. (C) Kaplan-Meier analysis of overall survival, according to *SMAD4* mRNA expression using publicly available gastric cancer microarray datasets (Kaplan-Meier Plotter). (D) Expression of *SPP1* mRNA in *HOXC8* knockdown cells compared with shluc control. (E) Relative mRNA levels of *SPP1* in stomach adenocarcinoma tissues (The Cancer Genome Atlas dataset). (F) According to *SPP1* mRNA expression to analysis overall survival, using publicly available gastric cancer microarray datasets (Kaplan-Meier Plotter). (G) Kaplan-Meier analysis of overall survival in patients with gastric cancer is achieved by examining the combination of *HOXC8* and *SPP1* mRNA levels (Kaplan-Meier Plotter). Other: low *HOXC8*/high *SPP1* and high *HOXC8*/low *SPP1*. (H) Comparison of OPN, pAKT, AKT, pERK, ERK, and β-actin levels between *HOXC8*-knockdown AZ521 cells and shluc control cells.

The *SPP1* gene contains a *HOXC8* responsive element in the promoter region 37. We also used the pathway commons database (https://www.pathwaycommons.org/) to identify the associated molecular targets of HOXC8. As show in Figure 4A, HOXC8 interaction with 24 genes, including SMAD4, PRDM4, GMNN, ZFP90, PBX2, RBPMS, PBX1, PDE4DIP, PLA2G10, ABL1, C1orf109, KRTAP12-1, LHX2, TEKT4, LHX3, TRIM42, FNTB, BLZF1, CYSRT1, SMAD1, KPRP, ZRANB1 and FOXO1. In this study, we focus on SMAD4 which has been reported to mediate OPN expression in cancer cells 38. We examined the correlation between *HOXC8* and *SMAD4* in patients with STAD using the TIMER. We found that *HOXC8* levels was significantly negatively correlated with *SMAD4* levels in STAD patients (Figure 4B). *SMAD4* upregulated level associated prolong overall survival in gastric cancer patients (Figure 4C). We further confirmed whether there was *HOXC8*-mediated *SPP1* expression in gastric cancer cells. The results showed that the expression of *SPP1* was significantly reduced in gastric cancer cells (Figure 4D). We then examined *SPP1* expression in gastric cancer patients using the TCGA database. *SPP1* was upregulated in STAD tissues compared to normal tissues (Figure 4E). Although *SPP1* expression has not significantly associated with overall survival in gastric cancer patients (Figure 4F). The high *HOXC8*/high *SPP1* group was associated with shorter overall survival than the low *HOXC8*/low *SPP1* group was among gastric cancer patients (Figure 4G). Moreover, OPN modulated the phosphorylation of AKT and ERK in lung cancer cells 39. *HOXC8* knockdown significantly reduced the phosphorylation of AKT and ERK in gastric cancer cells (Figure 4H).

## Discussion

In this study, the HOXC family was evaluated in patients with STAD, and *HOXC8* expression was upregulated in STAD tumor tissues and associated with worse overall survival. High levels of *HOXC8* mRNA expression also correlated with tumor stats, indicating that *HOXC8* plays a role in STAD progression. Although *HOXC8* reportedly mediate cell viability in gastric cancer cell lines 36, the role of *HOXC8* in gastric cancer has not been fully evaluated. We revealed that *HOXC8* knockdown reduced the expression of OPN and phosphorylation of AKT/ERK in gastric cancer cells.

Accumulating evidence shows that deregulated *HOX* gene expression promotes malignant transformation in many cancers, including leukemias, breast, cervical, ovarian, prostate, colorectal, melanoma, and squamous cell carcinoma. *HOX* genes reportedly have both oncogenic and tumor suppressing functions in cancer 25, 40, 41. Furthermore, *HOXC8* mediates cancer progression in multiple cancers, including lung, cervical, and gastric cancer 13, 36, 42. In our study, the *HOXC* family exhibited genetic alterations and co-occurrence in the same STAD patients. *HOXC8* was upregulated in STAD tissues and was associated with worse overall survival. High expression of *HOXC8* was associated with the tumor grades in STAD patients. Our data also revealed that *HOXC8* knockdown reduced the cell growth and colony formation ability in AZ521 and HR cells.

OPN is frequently observed in multiple human cancers, which contributes to tumor formation and progression. OPN expression is significantly upregulated in most gastric cancer patients at both the RNA and protein levels. Furthermore, OPN expression is significantly associated with a low apoptotic index, high proliferative index, low grade, high stage, lymph node and vascular invasion, and distal metastasis in the clinicopathology of gastric cancer patients 30, 43-47. An earlier study showed OPN as a direct target of *HOXC8* in C57BL/6J mouse embryo fibroblast cells. OPN was downregulated in *HOXC8*-overexpressed cells 37. Adwan *et al.* showed that *HOXC8* knockdown induced OPN expression in Suit2-007 cells 11. In our study, *HOXC8* knockdown significantly inhibited the cell growth and colony formation, and decreased the expression of OPN in gastric cancer cells. The high *HOXC8*/high *OPN* expression group was associated with shorter overall survival compared to that of the low *HOXC8*/high *OPN* expression group among gastric cancer patients. These findings suggest that the *HOXC8*/OPN axis may play a role in gastric cancer progression.

An earlier study reported that OPN is responsible for modulating the AKT and MAPK pathways and cell proliferation* in vitro* 48. In this study, we further evaluated the effect of *HOXC8* on the expression of OPN and the AKT/ERK pathway in gastric cancer cells. *HOXC8* knockdown significantly inhibited the expression of OPN and the phosphorylation of AKT/ERK in gastric cancer cell lines. We could not exclude the possibility that *HOXC8* directly or indirectly modulates OPN expression; however, this is the first study to evaluate alterations in *HOXC8* and OPN expression in gastric cancer cells.

## Conclusions

After evaluating the genetic alteration of the HOXC family, we focused on *HOXC8* and its upregulation was associated with the tumor grade in patients with STAD. This study is the first to investigate the molecular mechanism by which *HOXC8* modulates cell growth, colony formation, and the OPN-related pathway of gastric cancer cells. We found that *HOXC8* expression correlates with poor overall survival in gastric cancer patients, indicating that *HOXC8* is an independent prognostic factor.

## Figures and Tables

**Table 1:** Co-occurring of HOXC family genetic alterations in stomach adenocarcinoma.

A	B	Neither	A Not B	B Not A	Both	Log2 Odds Ratio	p-Value	q-Value	Tendency
HOXC5	HOXC10	1385	9	10	24	>3	<0.001	<0.001	Co-occurrence
HOXC4	HOXC8	1404	4	3	17	>3	<0.001	<0.001	Co-occurrence
HOXC4	HOXC6	1399	3	8	18	>3	<0.001	<0.001	Co-occurrence
HOXC5	HOXC9	1388	13	7	20	>3	<0.001	<0.001	Co-occurrence
HOXC6	HOXC8	1399	9	3	17	>3	<0.001	<0.001	Co-occurrence
HOXC8	HOXC9	1398	3	10	17	>3	<0.001	<0.001	Co-occurrence
HOXC4	HOXC9	1397	4	10	17	>3	<0.001	<0.001	Co-occurrence
HOXC9	HOXC10	1386	8	15	19	>3	<0.001	<0.001	Co-occurrence
HOXC5	HOXC8	1392	16	3	17	>3	<0.001	<0.001	Co-occurrence
HOXC4	HOXC11	1402	6	5	15	>3	<0.001	<0.001	Co-occurrence
HOXC4	HOXC5	1391	4	16	17	>3	<0.001	<0.001	Co-occurrence
HOXC4	HOXC10	1390	4	17	17	>3	<0.001	<0.001	Co-occurrence
HOXC6	HOXC9	1392	9	10	17	>3	<0.001	<0.001	Co-occurrence
HOXC11	HOXC12	1395	4	13	16	>3	<0.001	<0.001	Co-occurrence
HOXC10	HOXC11	1390	18	4	16	>3	<0.001	<0.001	Co-occurrence
HOXC8	HOXC10	1390	4	18	16	>3	<0.001	<0.001	Co-occurrence
HOXC6	HOXC11	1397	11	5	15	>3	<0.001	<0.001	Co-occurrence
HOXC8	HOXC11	1402	6	6	14	>3	<0.001	<0.001	Co-occurrence
HOXC12	HOXC13	1387	12	12	17	>3	<0.001	<0.001	Co-occurrence
HOXC5	HOXC6	1386	16	9	17	>3	<0.001	<0.001	Co-occurrence
HOXC6	HOXC10	1385	9	17	17	>3	<0.001	<0.001	Co-occurrence
HOXC4	HOXC12	1393	6	14	15	>3	<0.001	<0.001	Co-occurrence
HOXC5	HOXC13	1383	16	12	17	>3	<0.001	<0.001	Co-occurrence
HOXC10	HOXC13	1382	17	12	17	>3	<0.001	<0.001	Co-occurrence
HOXC9	HOXC11	1395	13	6	14	>3	<0.001	<0.001	Co-occurrence
HOXC8	HOXC12	1393	6	15	14	>3	<0.001	<0.001	Co-occurrence
HOXC8	HOXC13	1393	6	15	14	>3	<0.001	<0.001	Co-occurrence
HOXC11	HOXC13	1393	6	15	14	>3	<0.001	<0.001	Co-occurrence
HOXC6	HOXC12	1388	11	14	15	>3	<0.001	<0.001	Co-occurrence
HOXC4	HOXC13	1392	7	15	14	>3	<0.001	<0.001	Co-occurrence
HOXC10	HOXC12	1381	18	13	16	>3	<0.001	<0.001	Co-occurrence
HOXC5	HOXC11	1389	19	6	14	>3	<0.001	<0.001	Co-occurrence
HOXC6	HOXC13	1387	12	15	14	>3	<0.001	<0.001	Co-occurrence
HOXC9	HOXC12	1386	13	15	14	>3	<0.001	<0.001	Co-occurrence
HOXC9	HOXC13	1386	13	15	14	>3	<0.001	<0.001	Co-occurrence
HOXC5	HOXC12	1380	19	15	14	>3	<0.001	<0.001	Co-occurrence

**Table 2:** Association of *HOXC8* expression with clinicopathological characteristics in 367 gastric cancer patients.

			HOXC8			
			Low	High		
Variables	Item	Patient No.	No. (%)	No. (%)	p value*	
			126	241		
Sex	Female		41	148	0.304	
	Male		85	93		
Stage	I/II		57	110	1.000	
	III/IV		69	131		
T status	T1/T2		41	54	0.044*	
	T3/T4		85	187		
M status	Negative		118	223	0.831	
	Positive		8	18		
Lymph node status	Negative		36	81	0.347	
	Positive		90	160		

*P value < 0.05 was considered statistically significant (chi-square test for categorical variables).

## Abbreviations

HOX: homeobox
SPP1: secreted phosphoprotein 1
OPN: osteopontin
TCGA: The Cancer Genome Atlas
STAD: stomach adenocarcinoma
SMAD4: SMAD Family Member 4
PRDM4: PR Domain Zinc Finger Protein 4
GMNN: Geminin DNA Replication Inhibitor
ZFP90: ZFP90 Zinc Finger Protein
PBX2: PBX Homeobox 2
RBPMS: RNA Binding Protein, MRNA Processing Factor
PBX1: PBX Homeobox 1
PDE4DIP: Phosphodiesterase 4D Interacting Protein
PLA2G10: Phospholipase A2 Group X
ABL1: ABL Proto-Oncogene 1, Non-Receptor Tyrosine Kinase
C1orf109: Chromosome 1 Open Reading Frame 109
KRTAP12-1: Keratin Associated Protein 12-1
LHX2: LIM Homeobox 2
TEKT4: Tektin 4
LHX3: LIM Homeobox 3
TRIM42: Tripartite Motif Containing 42
FNTB: Farnesyltransferase, CAAX Box, Beta
BLZF1: Basic Leucine Zipper Nuclear Factor 1
CYSRT1: Cysteine Rich Tail 1
SMAD1: SMAD Family Member 1
KPRP: Keratinocyte Proline Rich Protein
ZRANB1: Zinc Finger RANBP2-Type Containing 1
FOXO1: Forkhead Box O1
